# A telomere-associated molecular landscape reveals immunological, microbial, and therapeutic heterogeneity in colorectal cancer

**DOI:** 10.3389/fmolb.2025.1615533

**Published:** 2025-05-26

**Authors:** Yinmeng Zhang, Jiawei Fan, Jiahui Zhao, He Zhu, Yan Xia, Hong Xu

**Affiliations:** Department of Gastroenterology, The First Hospital of Jilin University, Changchun, China

**Keywords:** colorectal cancer, telomere, microbiome, tumor microenvironment, prognosis

## Abstract

**Background:**

Colorectal cancer (CRC) ranks among the most prevalent malignancies of the gastrointestinal tract and remains a leading cause of cancer-related mortality worldwide. Although telomere biology has been increasingly implicated in immune modulation and tumor progression, its clinical significance in CRC remains poorly understood.

**Methods:**

We developed a telomere score, termed TELscore, by integrating transcriptomic and intratumoral microbiome profiles from publicly available colorectal cancer (CRC) cohorts. To comprehensively characterize TELscore subgroups, we performed pathway enrichment analysis, tumor immune microenvironment (TIME) profiling, and microbiome niche assessment. Whole-slide histopathological images (WSIs) and immunohistochemical (IHC) staining were utilized to visualize immune features, including tertiary lymphoid structures (TLSs), across subgroups. Patients were stratified into high and low TELscore categories, and the predictive robustness was validated across multiple independent training and validation cohorts. Chemotherapeutic drug sensitivity was evaluated using pharmacogenomic data from the Genomics of Drug Sensitivity in Cancer (GDSC) database. Furthermore, the predictive capacity of TELscore for immunotherapy response was independently assessed in an external cohort. Finally, single-cell RNA sequencing (scRNA-seq) analysis was conducted to further dissect the cellular landscape and immunological heterogeneity within the TME.

**Results:**

TELscore stratified patients into two biologically and clinically distinct subgroups. The high TELscore group, which exhibited significantly shorter DFS, showed marked enrichment of tumorigenic pathways such as EMT, along with a distinctly immunosuppressive TME. This was reflected by elevated ESTIMATE/TIDE scores and corroborated by CIBERSORT, which revealed increased infiltration of M0 macrophages and upregulation of immunosuppressive signatures. In contrast, the low TELscore group was enriched for cell cycle related pathways, including E2F targets and the G2/M checkpoint, and demonstrated higher infiltration of pro-inflammatory M1 macrophages. 16S rRNA sequencing further revealed a divergent intratumoral microbiome between subgroups, the high TELscore group harbored significantly greater relative abundance of Selenomonas and Lachnoclostridium, two pathogenic genera previously associated with colorectal tumorigenesis. Complementary histopathological assessment via WSI demonstrated a marked absence of intraTLSs in high TELscore tumors. From a therapeutic standpoint, high TELscore tumors exhibited reduced sensitivity to standard chemotherapeutic agents—including Fluorouracil, Irinotecan, Oxaliplatin, and Docetaxel—as reflected by elevated IC50 values. Conversely, these tumors demonstrated increased susceptibility to MAPK pathway inhibitors, such as Selumetinib and Trametinib. Notably, TELscore also served as a robust predictor of immunotherapy response, which was validated in the IMvigor210 cohort. Finally, scRNA analysis highlighted profound cellular and functional divergence between TELscore subgroups. We identified intensified intercellular communication between inflammatory macrophages and fibroblasts, reinforcing the presence of an immunosuppressive niche.

**Conclusion:**

TELscore is a robust stratification tool that captures the interplay between tumor biology, immune characteristics, and microbial ecology in colorectal cancer. By identifying clinically relevant subtypes with distinct therapeutic vulnerabilities, TELscore offers a powerful framework to advance personalized treatment and precision oncology.

## 1 Introduction

Colorectal cancer (CRC) ranks among the most prevalent malignancies of the gastrointestinal tract (Siegel et al., 2020). As reported by GLOBOCAN, CRC is the third most frequently diagnosed cancer worldwide and the second leading contributor to cancer-related mortality, with more than 1.9 million novel diagnoses recorded in 2022 (Bray et al., 2024). Currently, surgical resection remains the cornerstone of standard treatment for colorectal cancer (CRC). In addition to surgery, chemotherapy, often incorporating targeted agents, are routinely employed to improve clinical outcomes (Brenner et al., 2014). Although substantial improvements in early detection, surgical interventions, and chemotherapy have extended 5-year survival rates, roughly 30% of patients still face tumor recurrence after undergoing potentially curative surgery and postoperative therapy (Cheng et al., 2022). In addition, current systemic treatments, particularly immune checkpoint blockade, offer limited benefit to most CRC patients, highlighting the pressing demand for more reliable biomarkers to optimize individualized therapeutic strategies (André et al., 2020).

Telomeres, specialized nucleoprotein complexes at the ends of chromosomes, play a pivotal role in maintaining genomic integrity by preventing chromosomal termini from being mistaken as DNA damage or participating in end-to-end fusion events. Aberrations in telomere maintenance and dysregulation of telomerase, an enzyme that elongates telomeres, are widely recognized hallmarks of both aging and carcinogenesis (Mahfouz et al., 2017). In malignancies, approximately 85% of tumor cells overcome replicative senescence through telomerase reactivation or activation of the alternative lengthening of telomeres (ALT) pathway, thus acquiring unlimited proliferative potential (Shay, 2016). Beyond its canonical role, the telomerase reverse transcriptase (TERT) subunit has been shown to promote tumor progression through non-telomeric mechanisms, including activation of the PI3K/Akt pathway (Mahfouz et al., 2017) and modulating key regulators of cell cycle progression including Cyclin D1 and CDK4 (Samad et al., 2021). Interestingly, while telomere attrition and heterogeneity are frequently observed in colorectal and other cancers (Hastie et al., 1990), accumulating evidence suggests that maintenance of extended telomeres is essential for sustaining the proliferative drive of cancer cells (Shay, 2016; Günes and Rudolph, 2013). These paradoxical findings underscore the complexity of telomere dynamics in oncogenesis. Moreover, conventional measurements of average telomere length often yield inconclusive prognostic implications, likely due to tumor heterogeneity and methodological variability (Gao and Pickett, 2022), Therefore, a more integrative and multilayered approach is needed to clarify the role of telomere biology in CRC pathogenesis and progression.

In this study, we constructed a telomere-associated gene signature, termed the TELscore, by integrating bulk transcriptomic, 16S rRNA microbiome, and single-cell RNA sequencing data from over 2,113 CRC patients across multiple cohorts. The TELscore stratified patients into biologically and clinically distinct subgroups, which exhibited significant differences in hallmark pathway enrichment, immune infiltration patterns, and microbial composition. To further characterize tumor microenvironment (TME) heterogeneity, we analyzed whole-slide histopathological images (WSIs) and immunohistochemical (IHC) profiles, revealing distinct stromal and immune features across TELscore groups. We also assessed chemotherapeutic response profiles and validated the TELscore’s predictive performance for immunotherapy outcomes in an external cohort.

In conclusion, we leveraged single-cell transcriptomic data to dissect the intratumoral landscape, focusing on TELscore-related gene expression patterns across cell types and mapping cell–cell communication networks. Collectively, the TELscore demonstrates strong prognostic and predictive utility, providing mechanistic insights into telomere-associated pathways in CRC and offering a valuable tool to inform precision oncology.

## 2 Materials and methods

### 2.1 Data collection and preprocessing

A curated list of telomere-related genes was sourced from the TelNet database (http://www.cancertelsys.org/telnet/). Transcriptomic data and corresponding clinicopathological information were collected from multiple colorectal cancer (CRC) cohorts. The Cancer Genome Atlas CRC dataset (TCGA-CRC) was designated as the training cohort. Gene expression profiles and clinical follow-up data from seven independent Gene Expression Omnibus (GEO) datasets (GSE39582, GSE28722, GSE14333, GSE38832, and GSE41258) were used for external validation. To assess the immune landscape and predict immunotherapy response, single-cell RNA sequencing (scRNA-seq) data and clinical outcome information from 20 CRC patients were incorporated into the analysis. RNA-seq data for the TCGA-CRC cohort were downloaded from the UCSC Xena platform (Goldman et al., 2020), served as the primary training cohort, while clinical parameters were obtained from the Supplementary Material of Liu et al. (2018). For external validation, seven independent datasets (GSE39582, GSE28722, GSE14333, GSE38832, GSE41258) were retrieved from the Gene Expression Omnibus (GEO; https://www.ncbi.nlm.nih.gov/geo/) and processed using the “GEOquery” R package. Transcriptomic profiles (expressed as transcripts per million, TPM), intratumoral microbiome data, and survival data from the AC-ICAM cohort were obtained from Roelands et al. (2023). To evaluate the tumor immune contexture and immunotherapeutic responsiveness, single-cell sequencing data and corresponding clinical annotations for the 20 CRC patients were obtained from Chen et al. (2024). Immunohistochemical data for selected genes were retrieved from The Human Protein Atlas (https://www.proteinatlas.org) to visualize protein expression patterns at the tissue level.

### 2.2 Construction and validation of TELscore

To screen telomere-related genes (TRGs) with prognostic significance, we performed univariate Cox regression analysis using disease-free survival (DFS) as the outcome metric. To enhance the robustness of the results, the procedure was bootstrapped 1,000 times, each iteration involving random resampling of 80% of the TCGA-CRC dataset. Telomere-related genes that consistently exhibited significant associations with DFS across most iterations were retained for subsequent modeling. TCGA-CRC cohort served as the training dataset. To minimize overfitting risks inherent to high-dimensional transcriptomic data, LASSO (Least Absolute Shrinkage and Selection Operator) regression was employed, resulting in the selection of a concise panel of genes with strong prognostic relevance. Using the final gene set and corresponding LASSO-derived coefficients, a telomere-related score (TELscore) was computed for each sample using the formula, TELscore = ∑*i* = 1 Coefficient (TRGi) * Expression (TRGi). Patients across all cohorts were stratified into high and low risk groups based on an optimal threshold determined by the Youden index, enabling consistent classification and downstream validation.

### 2.3 Assessment of biological characteristics and immune microenvironment

To systematically assess the tumor microenvironment (TME) and immune infiltration landscape, we calculated gene signature scores using single-sample Gene Set Enrichment Analysis (ssGSEA) through the “GSVA” R package. Hallmark gene sets were obtained from the Molecular Signatures Database (MSigDB, h. all.v2023.1. Hs.entrez) (Subramanian et al., 2005; Liberzon et al., 2015). Additionally, we incorporated immune cell related gene signatures curated by Charoentong et al. (2017) were utilized to estimate the infiltration of specific immune cell populations within the TME. To further investigate tumor immune interactions and potential immunotherapy response, we performed functional enrichment and immune checkpoint-related analyses using the “IOBR” R package (Zeng et al., 2021a). The relative proportions of immune cell subsets were deconvoluted via the CIBERSORT algorithm (Yoshihara et al., 2013), providing a refined view of immune infiltration.

### 2.4 Immune infiltration assessment and tissue imaging

To independently quantify stromal and immune cell content within the tumor microenvironment (TME), we employed the ESTIMATE algorithm (Yoshihara et al., 2013), which also allowed us to evaluate key immunological signatures, including immune checkpoint gene expression, as well as markers of immune suppression, exclusion, and exhaustion. In addition, established indicators of immunotherapy response were examined, notably the Tumor Immune Dysfunction and Exclusion (TIDE) score (Jiang et al., 2018) and the tumor microenvironment (TME) score (Zeng et al., 2021b). Tertiary lymphoid structures (TLSs), which are ectopic lymphoid aggregates that emerge in non-lymphoid tissues during chronic inflammation or cancer, were also assessed. TLSs are typically composed of dense, unencapsulated clusters of CD20^+^ B cells, adjacent CD3^+^ T cell zones, and surrounding CD11c^+^ dendritic cells (Lei et al., 2024). To evaluate their prognostic relevance in colorectal cancer, a total of 598 whole-slide imaging (WSIs) from the TCGA-CRC cohort were manually annotated by board-certified pathologists blinded to clinical information. TLSs were stratified into peritumoral (periTLSs) and intratumoral (intraTLSs) categories based on their location relative to the invasive tumor front. To visualize the expression patterns of TELscore selected genes, immunohistochemistry (IHC) images from both tumor and adjacent normal tissues were retrieved from The Human Protein Atlas (https://www.proteinatlas.org/).

### 2.5 Microbial analysis, immunotherapy response and single cell analysis

Drug response predictions for conventional chemotherapeutic agents were obtained using the Genomics of Drug Sensitivity in Cancer (GDSC) database (Yang et al., 2013). To explore microbial variations within the tumor microenvironment, we analyzed 16S rRNA sequencing data from the AC-ICAM cohort to compare microbiome composition across different molecular subtypes (Roelands et al., 2023). Additionally, an independent immunotherapy dataset was incorporated to externally validate our findings. The IMvigor210 cohort, comprising patients with metastatic urothelial carcinoma treated with anti–PD-L1 therapy, was accessed using the “IMvigor210CoreBiologies” R package (Mariathasan et al., 2018). For single-cell level analysis, scRNA-seq data from Chen et al. (2024), were processed using the “Seurat” R package, adhering to the original study’s preprocessing protocols to ensure consistency and reproducibility. Intercellular communication and ligand–receptor interactions were inferred using the “CellChat” R package, enabling systematic reconstruction of signaling networks among diverse cell populations.

### 2.6 Statistical analysis

All computational analyses and statistical evaluations were conducted using R software (version 4.1.0). For two-group comparisons, we utilized the Wilcoxon rank-sum test, while multi-group comparisons were assessed using the Kruskal–Wallis’s test. Survival analyses were visualized via Kaplan–Meier curves, and statistical significance between groups was determined using the log-rank test through the “survminer” package. Correlations between variables were assessed using Spearman’s rank correlation method. Statistical significance was defined as a two-tailed p-value less than 0.05.

## 3 Results

### 3.1 Development and validation of TELscore

**FIGURE 1:**
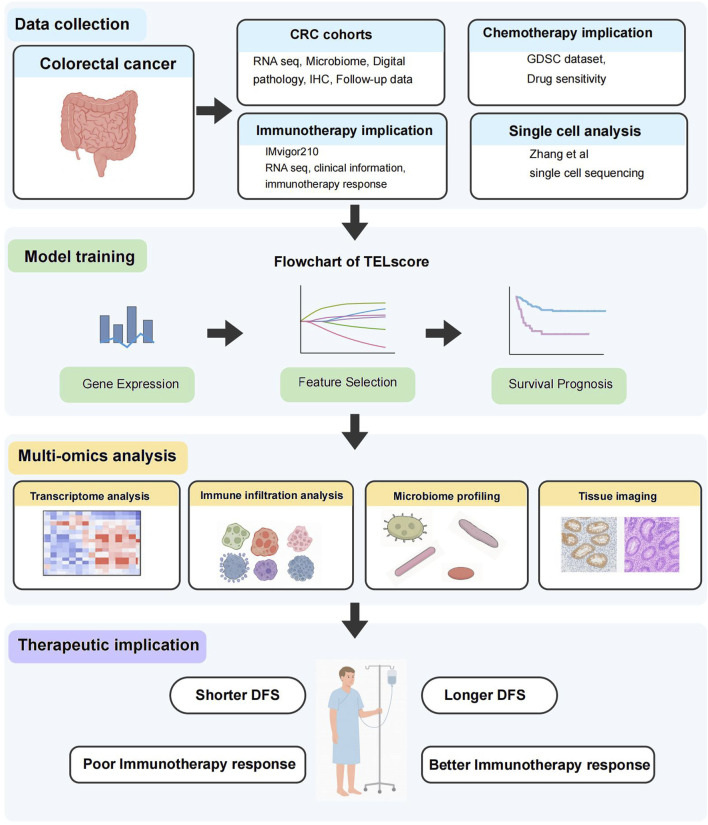
The overall workflow of this study.

**FIGURE 2:**
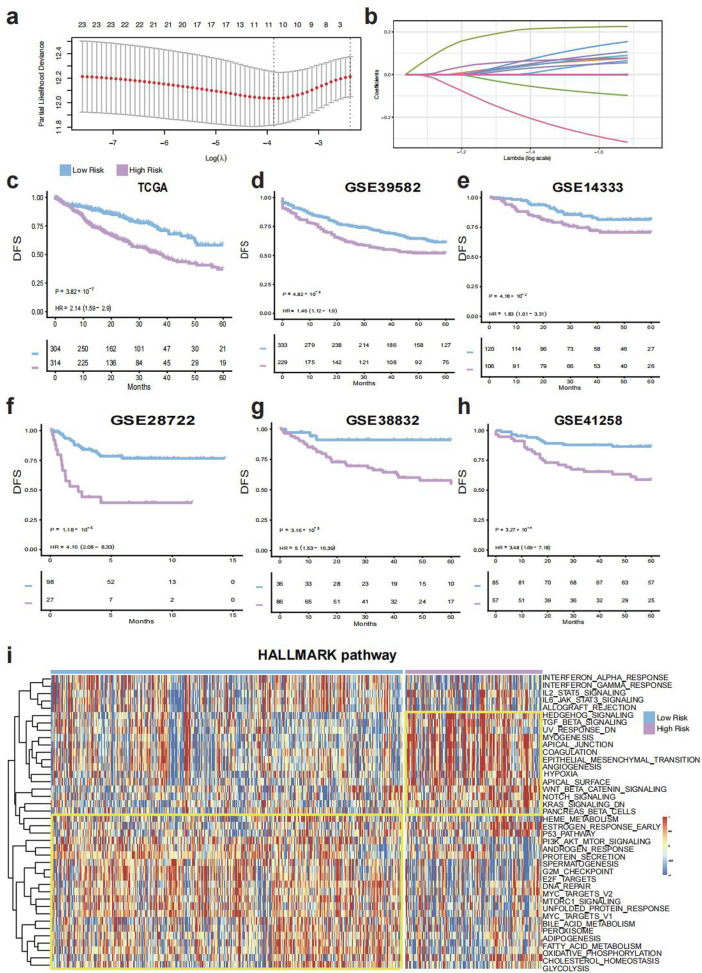
Construction and validation of the TELscore. **(a)**, Features selected by the LASSO Cox regression model. **(b)**, Selected genes and their corresponding coefficients. **(c)**, Kaplan–Meier survival analysis in the training cohort (TCGA-CRC). **(d–h)**, Survival validation in external cohorts: GSE39582, GSE14333, GSE28722, GSE38832, and GSE41258. **(i)**, Heatmap of Hallmark pathway enrichment between high and low TELscore groups.

The comprehensive workflow was shown in Figure 1. The telomere-related genes (TRGs) were acquired from TelNet (http://www.cancertelsys.org/telnet) (Braun et al., 2018), finally 25 genes were utilized in the construction of TELscore after the resampling process. A 11 gene signature was developed, and the TELscore formula is as follows: (0.076 * APOD) + (0.226 * CRY2) + (−0.098 * CXCL10) + (0.002 * GPX3) + (0.089 * NR4A1) + (0.065 * PTK7) + (0.154 * RNASE1) + (0.107 * SEZ6L2) + (0.055 * SLC2A1) + (0.077 * TIMP1) + (−0.317 * VWA5A) (Figures 2A,B). According to the optimal cut-off value, patients were classified into two TELscore group. Samples with higher TELscore trended to significantly shorter DFS in both training cohort and validation cohorts (Figures 2C–H). The high TELscore group exhibited a significantly higher enrichment in EMT, angiogenesis, hypoxia pathways, while G2M checkpoint and E2F targets were enriched in the low TELscore group (Figure 2I). Only pathways with statistically significant differences between the two groups (Wilcoxon test, adjusted p < 0.05) are shown in the heatmap. The functional annotations of the 11 core telomere-related genes constituting the TELscore are summarized in Supplementary Table S1, based on information retrieved from the NCBI Gene database (https://www.ncbi.nlm.nih.gov/gene/).

### 3.2 Molecular and immune characteristics of TELscore groups

**FIGURE 3:**
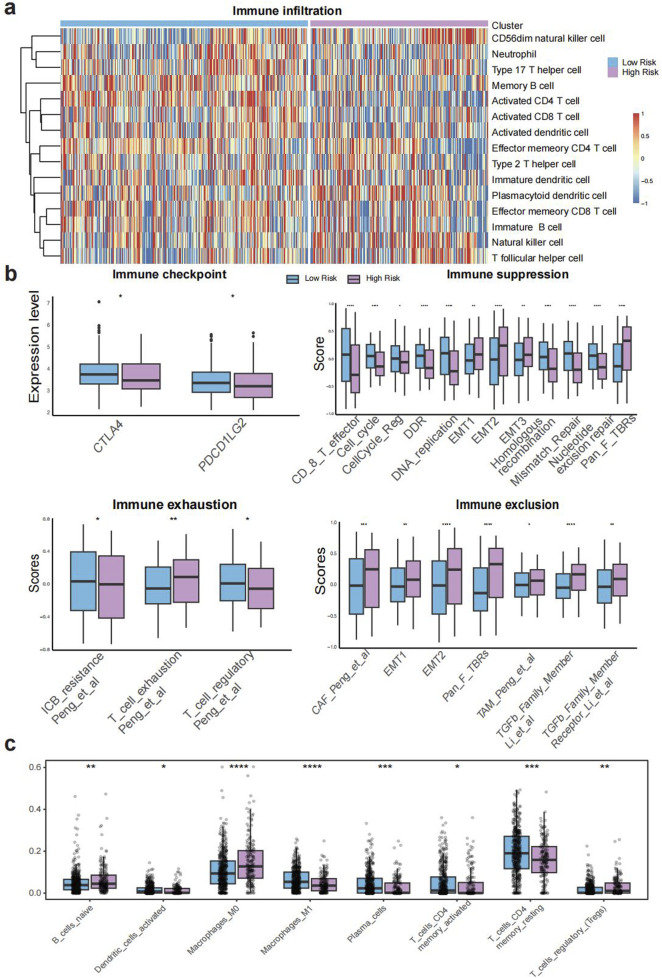
Molecular and immune characteristics associated with TELscore. **(a)**, Heatmap of immune cell infiltration across TELscore groups. **(b)**, IOBR-derived expression profiles of immune checkpoint genes, immunosuppressive markers, exhaustion signatures, and exclusion indicators. **(c)**, CIBERSORT based deconvolution analysis comparing immune cell compositions between high and low TELscore groups.

Both TELscore subgroups demonstrated extensive immune infiltration, as assessed using signatures derived from Charoentong et al. Notably, the high TELscore group exhibited significantly higher infiltration of CD56dim natural killer cells, natural killer cells, plasmacytoid dendritic cells, and T follicular helper cells compared to the low TELscore group (all p < 0.001; Figure 3A). Based on the IOBR algorithm, the high TELscore group exhibited elevated levels of immune exclusion, exhaustion, and immunosuppression, whereas the low TELscore group showed higher expression of immune checkpoint-related genes (Figure 3B). Furthermore, CIBERSORT-based deconvolution analysis revealed that the high TELscore group was characterized by increased infiltration of M0 macrophages and reduced presence of M1 macrophages within the tumor microenvironment (Figure 3C), indicating a shift toward an immunosuppressive phenotype.

### 3.3 Immune microenvironment and histopathological features of TELscore groups

**FIGURE 4:**
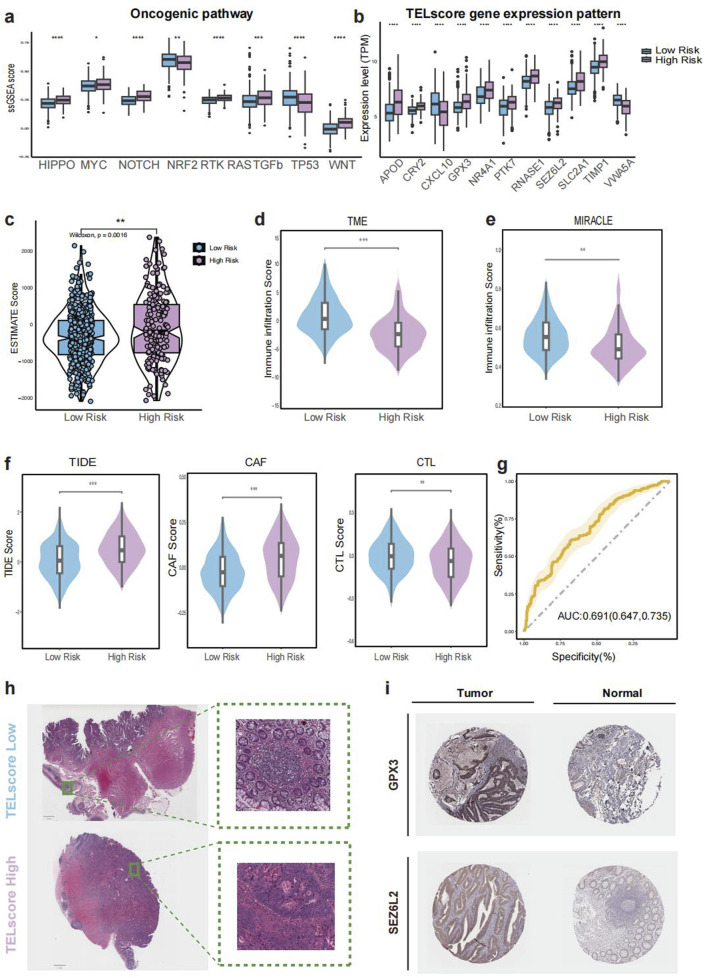
Immune microenvironment and histopathological features of TELscore groups. **(a)**, Boxplot comparing enrichment of oncogenic pathways between TELscore groups. **(b)**, ESTIMATE-derived stromal and immune scores across TELscore groups. **(c–g)**, TME-related scores and TIDE-based immunotherapy prediction scores across groups. **(h)**, Representative whole-slide imaging (WSI) for high and low TELscore tumors. **(i)**, Immunohistochemical (IHC) staining of selected TELscore features in normal versus tumor tissues.

The high TELscore group exhibited significantly elevated mutation enrichment in several key oncogenic pathways, including HIPPO, MYC, NOTCH, RTK-RAS, TGF-β, and WNT (Figure 4A), all of which are closely associated with tumor cell proliferation, invasion, and immune regulation. Additionally, the expression levels of genes comprising the TELscore model were significantly higher in the high TELscore group compared to the low TELscore group (Figure 4B). Consistently, patients in the high TELscore group demonstrated higher ESTIMATE scores, suggesting increased stromal and immune cell infiltration (Figure 4C). In contrast, the low TELscore group exhibited significantly higher TME and MIRACLE scores, indicative of a more favorable, immune-active tumor microenvironment (Figures 4D,E). The TIDE score, which reflects immune evasion potential, was also markedly elevated in the high TELscore group, in line with increased CAF infiltration and reduced cytotoxic T lymphocyte (CTL) abundance (Figure 4F). The prognostic power of the TELscore model was further supported by ROC curve analysis, yielding an AUC of 0.691 (95% CI: 0.647–0.735) for overall survival prediction (Figure 4G). whole-slide imaging (WSI) analysis from the TCGA-CRC cohort further supported these findings, revealing an absence of intraTLSs in the high TELscore group, whereas the low TELscore group displayed well-formed intraTLSs (Figure 4H). Additionally, TELscore signature genes such as GPX3 and SEZ6L2 were highly expressed in tumor tissues compared to adjacent normal tissues (Figure 4I).

### 3.4 Immunotherapy response, drug sensitivity, microbiome niche and single cell analysis

**FIGURE 5:**
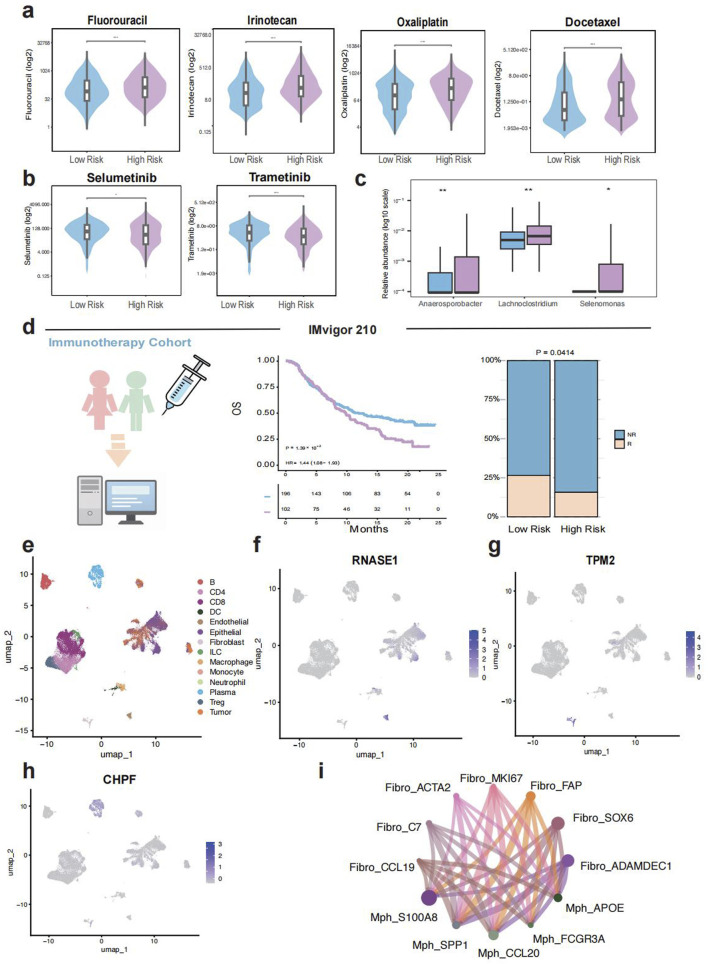
Immunotherapy response, drug sensitivity, microbiome niche and single cell analysis. **(a,b)**, Comparison of drug sensitivity (IC50 values) between different TELscore groups. **(c)**, Differential intratumoral microbiome composition based on 16S rRNA sequencing between high and low TELscore groups. **(d)**, Prognostic value and immunotherapy response prediction of TELscore in the IMvigor210 cohort. **(e–h)**, Single-cell transcriptomic analysis based on Zhang et al., including TELscore distribution and predicted immunotherapy responsiveness. **(i)**, Cell–cell communication networks inferred from single-cell data.

We evaluated the drug sensitivity of TELscore-defined subgroups using data from the Genomics of Drug Sensitivity in Cancer (GDSC) database (23,180,760). The high TELscore group exhibited significantly higher half-maximal inhibitory concentrations (IC50) for classical chemotherapeutic agents including Fluorouracil, Irinotecan, Oxaliplatin, and Docetaxel, suggesting reduced sensitivity to conventional chemotherapy (Figure 5A). In contrast, this group demonstrated lower IC50 values for Selumetinib and Trametinib, indicating that these agents may serve as promising therapeutic alternatives for patients with high TELscores (Figure 5B). Additionally, analysis of 16S rRNA microbiome data revealed a significantly higher relative abundance of potentially pathogenic genera, Anaerosporobacter, Lachnoclostridium, and Selenomonas, in the high TELscore group (Figure 5C). Evaluation of the IMvigor210 immunotherapy cohort further supported the prognostic relevance of TELscore, patients in the high TELscore group exhibited shorter overall survival and a significantly greater proportion of non-responders to treatment (Figure 5D). To further characterize the tumor microenvironment (TME), we analyzed single-cell transcriptomic data from Zhang et al. and identified 14 distinct cell types for downstream analysis (Figure 5E). Among TELscore-associated genes, RNASE1 was predominantly expressed in endothelial and epithelial cells, TPM2 in fibroblasts, and CHPF in plasma cells (Figures 5F–H). Furthermore, cell–cell communication analysis revealed enhanced interactions between fibroblasts and macrophages, particularly between CCL19^+^ fibroblasts and CCL20^+^ macrophages, as well as between S100A8^+^ macrophages and ADAMDEC1^+^ fibroblasts (Figure 5I).

## 4 Discussion

Colorectal cancer (CRC) remains one of the most prevalent and lethal malignancies worldwide, with over 1.9 million new cases and approximately 940,000 deaths reported in 2020. Global incidence is projected to rise to 3.2 million new cases by 2040 (Mangone et al., 2022). While the 5-year overall survival (OS) rate exceeds 90% for patients diagnosed under stage II, it declines sharply to below 25% in those with metastatic or advanced-stage CRC (Fang et al., 2021). Despite significant therapeutic advancements that have improved overall survival (OS), a considerable proportion of CRC patients still experience recurrence or resistance to chemotherapy, underscoring the urgent need for refined prognostic tools and therapeutic guidance (Rothwell et al., 2023). Telomerase reactivation, a hallmark of cancer, not only maintains telomere length via its core components TERT and TERC, but also exerts non-canonical mitochondrial functions—reducing ROS, DNA damage, and apoptosis (Liu et al., 2023; Jaiswal et al., 2013). Recent studies reveal that, dysfunction of telomere suppresses PGC in a p53-dependent manner, thereby increasing reactive oxygen species (ROS) levels, and impairing overall metabolic function (Amano et al., 2019). However, the precise role of telomere dynamics in CRC remains incompletely understood. Thus, developing a telomere-based prognostic model could offer new insights into tumor biology and assist in personalized clinical management.

In this study, we constructed a robust telomere-based scoring system, the TELscore, by integrating transcriptomic, microbiome, and single-cell RNA-seq data from over 2,113 CRC patients across multiple centers. The TELscore, consisting of eleven telomere-associated genes, exhibited strong prognostic performance in the training cohort (TCGA-CRC) and was independently validated across five external GEO cohorts. Patients with high TELscores displayed significant enrichment of tumorigenic signaling pathways, including epithelial-mesenchymal transition (EMT), angiogenesis, and inflammation-related signatures. Using data from Charoentong et al. (2017), we observed substantial immune cell infiltration in both TELscore subgroups. However, further immune profiling via the IOBR algorithm revealed that the high TELscore group exhibited elevated immune suppression, T-cell exclusion, and exhaustion scores, while the low TELscore group expressed higher levels of immune checkpoint genes.

CIBERSORT-based deconvolution further highlighted that the high TELscore group was characterized by increased infiltration of naïve B cells, regulatory T cells (Tregs), and M0 macrophages, but reduced M1 macrophage presence, indicating an immunosuppressive microenvironment in which macrophages remain in an undifferentiated M0 state without effective polarization toward the pro-inflammatory M1 phenotype (Wang et al., 2023). Additionally, we observed significant enrichment of multiple oncogenic pathways—including HIPPO, MYC, NOTCH, RTK-RAS, TGF-β, and WNT—in the high TELscore group, whereas TP53-related tumor-suppressive signaling was more prevalent in the low TELscore group (Kastenhuber and Lowe, 2017). Consistent with these findings, the high TELscore group also demonstrated higher TIDE and ESTIMATE scores, further supporting the presence of a suppressive tumor microenvironment (TME) and suggesting impaired responsiveness to immune checkpoint blockade (Qin et al., 2023).

Given the emerging role of tertiary lymphoid structures (TLSs) in orchestrating anti-tumor immunity, we evaluated their presence using whole-slide imaging (WSI) from the TCGA cohort. We observed a marked absence of intra-tumoral TLSs in the high TELscore group, which may contribute to poor immunotherapy response and adverse prognosis (Lei et al., 2024; Lynch et al., 2021). Immunohistochemical validation confirmed higher expression of key TELscore genes, including GPX3 and SEZ6L2, in tumor tissues compared to adjacent normal mucosa (Barrett et al., 2013; Zhang et al., 2025).

To assess potential clinical utility, we evaluated the predicted drug sensitivity profiles between TELscore subgroups. The high TELscore group showed significantly higher IC50 values for standard chemotherapeutic agents such as fluorouracil, irinotecan, oxaliplatin, and docetaxel, suggesting a diminished response to conventional therapies. Interestingly, this group exhibited markedly lower predicted IC50 values for MEK inhibitors Selumetinib and Trametinib, implicating these agents as promising alternatives for targeted therapy (Spreafico et al., 2013; Tian et al., 2023). Recent studies have increasingly highlighted the potential role of the gut microbiome in regulating host telomere dynamics (Pepke et al., 2024). Certain microbial taxa have been associated with longer telomeres and enhanced antioxidative capacity, suggesting that alterations in the gut microbiota may not only be a consequence of telomere dysfunction but could also act as a contributing factor (Velando et al., 2021). Emerging evidence supports a bidirectional interaction between telomere attrition and microbiota dysbiosis. For example, El Maï et al. demonstrated that telomerase-deficient zebrafish exhibited shortened telomeres, elevated DNA damage, and reduced microbial diversity, whereas gut-specific activation of telomerase partially reversed these effects (El Maï et al., 2023). Similarly, studies in telomerase-deficient mice revealed impaired intestinal barrier integrity and microbiome alterations linked to inflammation and oxidative stress (Chakravarti et al., 2020). These findings imply that the microbial shifts observed in high-TELscore CRC patients may not merely be downstream consequences of telomere dysfunction, but could also actively exacerbate genomic instability, immune dysregulation, and tumor progression through reciprocal host–microbiota interactions. Therefore, we further explored the microbiome landscape using 16S rRNA intratumoral sequencing data from the AC-ICAM cohort. The high TELscore group harbored increased abundance of the pathogenic genus Selenomonas, along with enrichment of Lachnoclostridium, a proposed marker for colorectal adenomas and Anaerosporobacter, whose role in CRC remains undefined (Bullman et al., 2017; Yang et al., 2023; Liang et al., 2020). These microbial alterations may contribute to the immune dysfunction and unfavorable prognosis observed in this subgroup. Validation in an independent immunotherapy cohort, IMvigor210, confirmed that patients in the high TELscore group had poorer clinical outcomes and a significantly higher proportion of non-responders, supporting the predictive value of the TELscore for immunotherapeutic efficacy. Lastly, we performed single-cell RNA-seq analysis to further investigate the expression landscape and cell–cell interactions associated with TELscore-related genes. We found that genes such as RNASE1, TPM2, and CHPF were highly expressed in endothelial cells, fibroblasts, and plasma cells. Moreover, inflammatory macrophage subsets, including S100A8+ and CCL20+ macrophages exhibited enhanced communication with ADAMDEC1+ and SOX6+ fibroblasts, potentially contributing to the immunosuppressive in the high TELscore group.

In summary, this study integrated multi-omics data to construct TELscore, a novel and robust scoring system for stratifying CRC patients and informing clinical decision-making. Through comprehensive characterization of telomere related features including molecular signatures, immune infiltration patterns, microbiome composition, pathological subtypes, and immunohistochemical profiles, TELscore reflects the immunological heterogeneity within CRC. While our findings provide important insights, several limitations should be acknowledged. First, as a retrospective, multi-center analysis, prospective clinical validation is necessary to confirm the prognostic and therapeutic relevance of telomere-related features. Second, this study lacks experimental validation using tissue samples. Future work will focus on validating the TELscore-associated molecular and immunological characteristics through independent patient cohorts and functional experiments to enhance its translational potential. Third, due to the limited availability of publicly recognized CRC immunotherapy cohorts, we used the widely accepted IMvigor210 dataset to assess the predictive value of TELscore. Future studies incorporating CRC-specific immunotherapy cohorts are warranted to further validate the utility of TELscore in predicting treatment response.

Altogether, our TELscore framework offers a valuable tool for predicting patient prognosis and response to immunotherapy and highlights the multifaceted role of telomere in CRC progression and clinical management.

## 5 Conclusion

This study established and validated a robust TELscore system for quantifying CRC patients and systematically elucidated the role of telomere in tumor biology, immune microenvironment, and microbial compositions. The predictive value of clinical prognosis, immunotherapy response and drug sensitivity, was confirmed in independent cohorts. These findings, further supported by pathological and immunohistochemical evidence, provide novel insights to guide precision clinical management in colorectal cancer.

## Data Availability

The datasets presented in this study can be found in online repositories. The names of the repository/repositories and accession number(s) can be found in the article/Supplementary Material.
