# Supplementary material for: A telomere-associated molecular landscape reveals immunological, microbial, and therapeutic heterogeneity in colorectal cancer
Source: Front Mol Biosci. 2025 May 26;12:1615533. doi: 10.3389/fmolb.2025.1615533 (PMC12146184; doi:10.3389/fmolb.2025.1615533)
Supplement: Supplementary file 1 [file Table1.docx]

| **Gene Symbol** | **Description** |
| --- | --- |
| [APOD](https://www.ncbi.nlm.nih.gov/gene/347) | This gene encodes a component of high density lipoprotein that has no marked similarity to other apolipoprotein sequences. |
| CRY2 | This gene encodes a flavin adenine dinucleotide-binding protein that is a key component of the circadian core oscillator complex, which regulates the circadian clock. |
| CXCL10 | This antimicrobial gene encodes a chemokine of the CXC subfamily and ligand for the receptor CXCR3. |
| GPX3 | The protein encoded by this gene belongs to the glutathione peroxidase family, members of which catalyze the reduction of organic hydroperoxides and hydrogen peroxide (H2O2) by glutathione, and thereby protect cells against oxidative damage. |
| NR4A1 | This gene encodes a member of the steroid-thyroid hormone-retinoid receptor superfamily. Expression is induced by phytohemagglutinin in human lymphocytes and by serum stimulation of arrested fibroblasts. |
| PTK7 | This gene encodes a member of the receptor protein tyrosine kinase family of proteins that transduce extracellular signals across the cell membrane. |
| RNASE1 | This gene encodes a member of the pancreatic-type of secretory ribonucleases, a subset of the ribonuclease A superfamily. |
| SEZ6L2 | This gene encodes a seizure-related protein that is localized on the cell surface. |
| SLC2A1 | This gene encodes a major glucose transporter in the mammalian blood-brain barrier. |
| TIMP1 | This gene belongs to the TIMP gene family.The proteins encoded by this gene family are natural inhibitors of the matrix metalloproteinases (MMPs), a group of peptidases involved in degradation of the extracellular matrix. |
| VWA5A | This gene located in nucleoplasm. |

**Supplementary Table 1.TELscore Gene Functional Annotations.**
